# Our Contemporaries

**Published:** 1916-04

**Authors:** 


### OUR CONTEMPORARIES.

Health News, the Bulletin of the N. Y. State Dept. of Health, devotes its March issue to cancer. Every physician should read this number carefully, noting especially that the State Institute for the Study of Malignant Diseases will examine specimens free of charge.

The Proctologist appears, beginning with the March issue, as The Proctologist and Gastroenterologist, and will be published quarterly as the component journals have been. Dr. Rollin H. Barnes of St. Louis continues as managing editor and publisher. The editorial staff consists of Dr. Lewis Brinton, Philadelphia, Gastroenterology; Dr. Anthony Bassler, New York, same; Dr. A. L. Benedict, Buffalo, Dietetics. We may add that the last appointment is provisional and depends upon the ability to add to the amount of labor beyond practice.

---

The Medical Record celebrated its 50th anniversary in a special number of unusual excellence, March 4. Our congratulations to our young colleague.

---

The Journal of Immunology appears with Feb. 1916 and will be issued bi-monthly as the official organ of the Society for Serology and Haematology and the Am. Assn. of Immunologists. Dr. Arthur F. Coca will be at the head of the editorial staff of 36 members, there being also an international advisory board of nine: Simon Flexner, Wm. H. Park and Theobald Smith of N. Y.; Ludwig Hektoen of Chicago; Robert Muir of Glasgow; F. Nuttall of Cambridge; Victor C. Vaughan of Ann Arbor; Wm. H. Welch of Baltimore and A. E. Wright of London. It is unfortunate that this journal should perpetuate so abominable a word as Immunology but, otherwise, it is of the highest order. Since the death of Dr. Achilles Rose, we are at a loss when onamatologic reform is needed, but we suggest Amynology. Our best wishes to the new journal.

